# Serious Illness Communication in Emergency Medicine: A Badge Card for Practice and Teaching

**DOI:** 10.1002/aet2.70252

**Published:** 2026-09-10

**Authors:** Margaret Putman

**Affiliations:** ^1^ Section of Geriatrics & Palliative Medicine The University of Chicago Pritzker School of Medicine Chicago Illinois USA

**Keywords:** difficult conversations, goals of care, serious illness communication

Emergency physicians regularly encounter patients with life‐limiting illness who need support navigating complex medical decisions. Serious illness communication (SIC) to elicit preferences, clarify values, and promote shared decision making is integral to emergency medicine (EM) practice, and the American Board of Emergency Medicine (ABEM) 2026 certifying exam now includes dedicated communication cases [1].

Existing SIC frameworks including REMAP and the Serious Illness Conversation Guide were originally designed for established relationships and scheduled conversations [2, 3]. EM‐specific conversation guides exist but function as detailed protocols for specific clinical scenarios [4]. This two‐sided badge card distills evidence‐based SIC frameworks [2, 3, 4] into a portable clinical and teaching tool for any SIC, aligned with EM palliative care competencies [5, 6]. (Figure 1).

**FIGURE 1:**
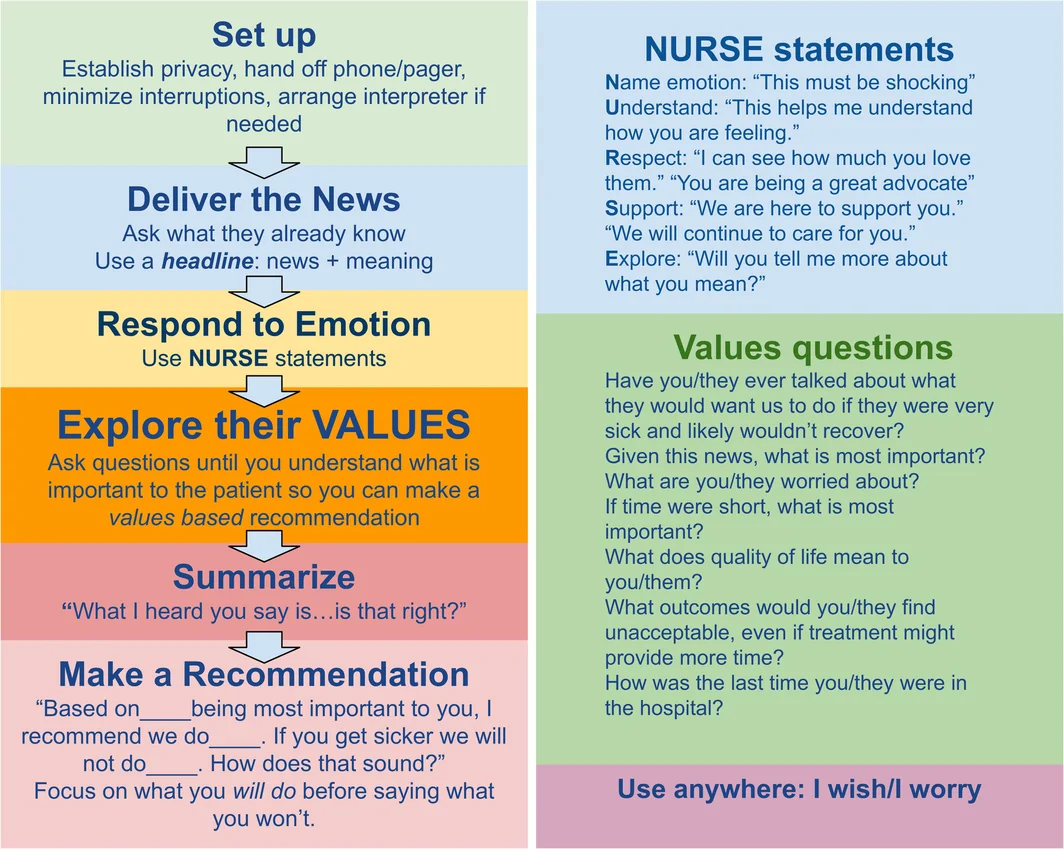
Serious Illness Communication Badge Card. Front side depicts the SIC framework (left); back side includes NURSE statements, values questions, and “I wish/I worry” language (right).

The framework is described below:

Before delivering difficult news, ask the patient or family what they already know [7]. This orients the clinician and identifies knowledge gaps (“What have you been told about your illness?”).

Deliver the news using a headline—a concise statement pairing the medical information with its meaning for the patient [2]. This should be free of jargon and include the impact on the patient's life. (“Your dad has a serious infection and his organs are shutting down. I'm worried he is dying.”).

Respond to emotion using NURSE statements (Naming, Understanding, Respecting, Supporting, Exploring) [7]. After delivering difficult news, the first response from the patient/family is likely emotion, even if it sounds like a cognitive statement or question. Respond to the underlying emotion first, as it is difficult to process information in a heightened emotional state.

Explore values by asking questions until you understand what matters most to the patient. “I wish/I worry” is language that can be used anywhere in the conversation: to align with the patient while delivering difficult news honestly, to respond when someone says “there must be more you can do,” and to bridge into values exploration [3, 7] (“I wish things were different. I worry that if we don't talk about this now, we won't be prepared.”).

Summarize before recommending. Reflect back what you heard and confirm before moving forward (“I heard you say… is that right?”).

Make a recommendation grounded in the patient's values. Lead with what you will do before saying what you won't [2, 3].

The card has been distributed at national conference events (AAEM Scientific Assembly, 2023) and incorporated into didactic curricula at multiple institutions, though formal evaluation of its impact has not been conducted. Before a family meeting, the learner can review the card and identify one skill to practice; afterward, the card provides shared language for targeted debriefing. It is equally useful as a clinical reference—review it before entering the room or keep it accessible during the encounter.

This tool synthesizes concepts from published SIC frameworks with language for EM use. As programs prepare for the ABEM certifying exam, this badge card provides a ready‐to‐use tool that requires no special resources, supports the conversations patients need, and prepares learners for SIC in independent practice.

## Author Contributions

**Margaret Putman:** conceptualization, writing – original draft, writing – review and editing.

## Funding

The author has nothing to report.

## Conflicts of Interest

The author declares no conflicts of interest.

## Data Availability

Data sharing not applicable to this article as no datasets were generated or analysed during the current study.
